# Associations between new health conditions and healthcare service utilizations among older adults in the United Kingdom: effects of COVID-19 risks, worse financial situation, and lowered income

**DOI:** 10.1186/s12877-022-02995-8

**Published:** 2022-04-22

**Authors:** Bingxue Han, Hongyi Guan

**Affiliations:** 1 International Issues Center, Xuchang University, Xuchang, Henan China; 2 Family Issues Center, Xuchang University, Xuchang, Henan China; 3 Xuchang Urban Water Pollution Control and Ecological Restoration Engineering Technology Research Center, Xuchang University, Xuchang, China; 4 College of Urban and Environmental Sciences, Xuchang University, Xuchang, China; 5 Grade 6 Class 7, Xuchang Municipal Xingye Road Primary School, Xuchang, Henan China

**Keywords:** COVID-19 risks, Treatment cancellation, Accessible care, Worse financial situation, Lowered income, New health conditions, Cross-sectional mediation, Cross-sectional moderation, Longitudinal mediation, Longitudinal moderation

## Abstract

**Background:**

Health services are critically important for older adults, particularly during the Coronavirus disease-19 (COVID-19) pandemic. However, COVID-19 risks, worse financial situation, and lowered income may seriously impact health services by feasibility and accessibility. Therefore, the aim of the present study was empirically to explore how health-seeking behaviors are influenced by new health conditions through COVID-19 risks, worse financial situation, and lowered income.

**Methods:**

Data were from ELSA COVID-19 waves 1 and 2 which included a sample of 6952 and 6710 older adults in the United Kingdom, respectively. The frequency distribution analyses were conducted by Chi-square analysis by gender groups. Zero-inflated Poisson regressions were used to examine how worse financial situation and lowered income were associated with COVID-19 risks and new health conditions. Logistic regressions were employed to examine the associations of COVID-19 risks, worse financial situation, and lowered income with treatment cancellation and accessible care. Cross-sectional mediation models, cross-sectional moderation models, longitudinal mediation models, and longitudinal moderation models were conducted based on Hayes model 6, Hayes model 29, Montoya model 1, and Montoya model 2, respectively.

**Results:**

Most of the sample was >65 years old, females, located in urban place, and involved in long-standing condition. Regression analysis showed that COVID-19 risks, worse financial situation, and lowered income were associated with treatment cancellation and accessible care. In the longitudinal mediations, effect coefficients of ‘X’ → (treatment cancellation in wave 1 (Tcn1)- treatment cancellation in wave 2 (Tcn2))(β = −.0451, *p* < .0001, low limit confidence interval (LLCI) = −.0618, upper limit confidence interval (ULCI) = −.0284), ‘X’ → (COVID-19 risks in wave 1 (Csk1)- COVID-19 risks in wave 2 (Csk2)) (β = .0592, *p* < .0001, LLCI = .0361, ULCI = .0824), and ‘X’ → (lowered income in wave 1 (CIn1)- lowered income in wave 2 (CIn2)) (β = −.0351, *p* = .0001, LLCI = -.0523, ULCI = -.0179) were significant. Additionally, effect coefficients of ‘X’ → (accessible care in wave 1 (Acr1)- accessible care in wave 2 (Acr2)) (β = .3687, *p* < .0001, LLCI = .3350, ULCI = .4025),'X’ → (Csk1- Csk2) (β = .0676, *p* = .0005, LLCI = .0294, ULCI = .1058), and ‘X’ → (worse financial situation in wave 1- worse financial situation in wave 2) (β = −.0369, *p* = .0102, LLCI = -.0650, ULCI = -.0087) were significant.

**Conclusions:**

There were longitudinal mediating effects of COVID-19 risks, worse financial situation, and lowered income on the relationship between new health conditions and treatment cancellation and relationship between new health conditions and accessible care. These findings suggest that worse financial situation, lowered income, and COVID-19 risks exerted an influence on the relationship between new health conditions and treatment cancellation and relationship between new health conditions and accessible care among older adults. Findings suggest that longitudinal mediations may be important components of interventions aiming to meet service needs. Long-term health policy implications indicate the need for reducing COVID-19 risks, improving financial situation, and increasing income among the targeted population.

**Supplementary Information:**

The online version contains supplementary material available at 10.1186/s12877-022-02995-8.

## Background

Selected health conditions often professionally correspond to likelihood of improvement with treatment and care. But, the prevalence of co-occurring psychiatric disorders, co-existing chronic health conditions, and concurrent behavioral health conditions often was high when diagnosed a specific disease [1, 2]. Thus, treatment challenges [3], treatment utilization [4], and treatment needs [5], and treatment effectiveness [6] were reported in such settings. Subsequently, patients’ perception regarding medication effectiveness could strongly lead to treatment satisfaction [7] and barriers for treatment-seeking [8]. Objectively, self-reports of treatment for secondary health conditions [9], affordable care [10], and mental health problems [11] were identified as potential predictors of treatment. Moreover, co-occurring health conditions often need collaborative care in the treatment [12] and integrating treatment [13]. Empirically, the association between health conditions demonstrates the need to consider in the context of other health hazards or different healthcare settings [14]. Early studies documented health-seeking behaviors among the persons with poor health conditions. For example, a cross-sectional survey in Albania concluded public facilities were reported as the main health care service providers by adults with non-communicable diseases [15]. An Australian cohort demonstrated that persons with hypertension and poor self-rated health were likely to visit frequent monthly general practitioners [16]. An observational study indicated improved healthcare access led to improved physical and mental health [17]. Thus, persons with health conditions may behave as health-seeking behaviours.

Factors including stigma [18], treatment costs [19], patient views [20], and health consultant [21] possibly lead to under-treatment of health conditions. In addition, socioeconomic disparities were identified in the reporting of health conditions and the use of discretionary health services [22]. In addition, a study indicated that uninsured individuals in poor health conditions consumed less health care [23]. Dynamic treatment regimes [24], personalized care [25], and accessible personal medicine [26] are considered as ideological and effective methods to manage and improve health status. Patients with acute health conditions were more likely to require hospitalization than those without. Collaborative care models [27] and interdisciplinary multi-professional treatment [28] are valuable approaches for patients with psychiatric and physical health conditions in real-life hospital settings. It was confirmed that web-based self-care interventions for chronic conditions could not be replaced with outpatient and inpatient treatment to reduce health inequalities [29].

Regarding early impacts of the COVID-19 pandemic on mental disorders, a review found deteriorations in symptoms, lack of access to services and resources, and self-management in inpatient and residential settings [30]. It was confirmed that the fear of COVID-19 could trigger preventive health behaviors [31]. In the time of COVID-19, persons with outdoor activities appear to be more susceptible to the coronavirus disease than before. During the Coronavirus disease-19 (COVID-19) period, a lot of studies reported change of health-seeking behaviors caused by COVID-19. Multiple studies identify the healthcare and emotional needs [32], use of new technology for social connectedness [33], accessibility and utilisation of telehealth [34] among older adults during the COVID-19 pandemic. Empirical studies have reported self-cancelled appointments [35], health-check cancellation [36], cancelled surgeries [37], canceled appointments [38], treatment pause [39], therapy postponements [40, 41], fertility treatment cancellation [42] due to COVID-19 pandemic. Delay in fertility treatment [43], fertility treatment suspensions [44], and cancelled appointments [45] were also reported during COVID-19 pandemic. Thus, COVID-19 pandemic may significantly influence utilizations of health care service of older adults.

In this context, the goal of this study was to explore the mediating factors with ELSA COVID-19 with data collected in representative samples of UK, in 2021. The main hypothesis in this study was that the relationships of new health conditions → treatment/care would be mediated/moderated by barriers to accessing health services. Here, barriers to accessing health services included COVID-19 risks, worse financial situation, and lowered income.

### Theory and hypothesis

The main relationships of interest were motivated on the basis of Health Belief Model (HBM). HBM empowers researchers to explain and predict health promoting behaviour in terms of patterns of belief by addressing the association between personal threat of an illness or disease and health services utilisation [46–48]. Facing the susceptible risk of COVID-19 pandemic, the public population with new health conditions would like to perform care/treatment-seeking behaviors when considering the related severity and barriers.

In this study, mediators /moderators included COVID-19 risks, worse financial situation, and lowered income. A large body of research documented economic burden and needs of health care service. For example, a study indicated that COVID-19 lockdown reduced numbers of patients living in poor socio-economic conditions accessing the mental health service [49]. A study among rural migrant workers in China indicated that unequal health service utilization sourced from income-related inequality [50]. A study in Japan concluded change in household income might influence the utilization of long-term home care services [51]. Simultaneously, multiple studies documented financial barriers to access to health services [52–55]. Forgoing necessary medical treatments [56] and refraining from health care [57] were reported. Financial hardship and health risk behavior were reported during COVID-19 in a large US national sample of women [58]. A study in among African American men suggested that financial hardship can lead to unmet medical need due to cost [59]. A study in central Malawi indicated financial accessibility of health services was identified the determinants of health seeking behaviours [60]. Several studies indicated part of patients did not seek further treatment testing due to low socio-economic status [61, 62]. Socio-economic factors regarding early signs and symptoms resulted in delayed diagnosis [63]. Furthermore, the general population is experiencing more new health conditions than ever given the current COVID-19 pandemic. COVID-19 has been confirmed as a significant impact on the quality of life, general health, and social relationships, and illness acceptance of patients [64].

**Fig. 1:**
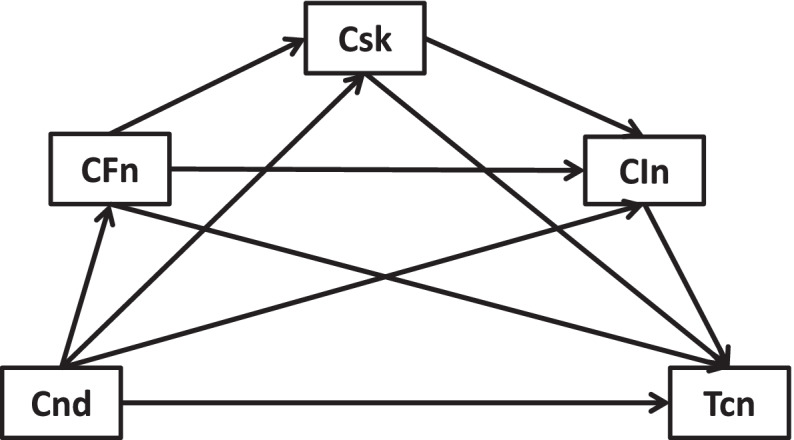
Cross-sectional link of new health conditions →treatment cancellation mediated by COVID-19 risks, worse financial situation, and lowered income

**Fig. 2:**
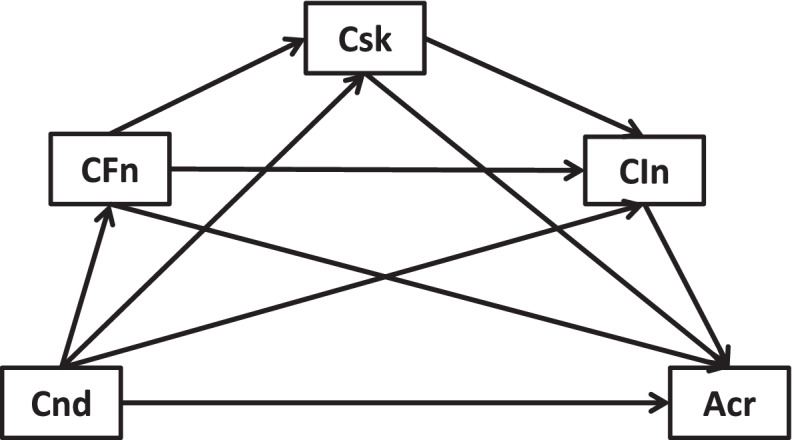
Cross-sectional link of new health conditions →accessible care mediated by COVID-19 risks, worse financial situation, and lowered income

**Fig. 3:**
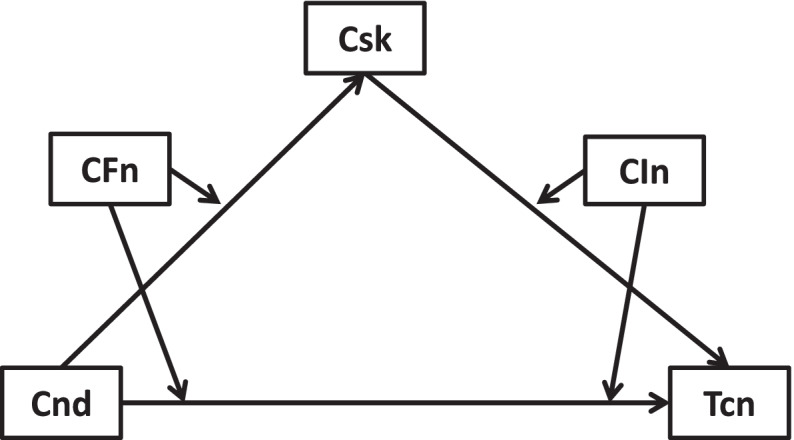
Cross-sectional link of new health conditions →treatment cancellation mediated by COVID-19 risks and moderated by worse financial situation and lowered income

**Fig. 4:**
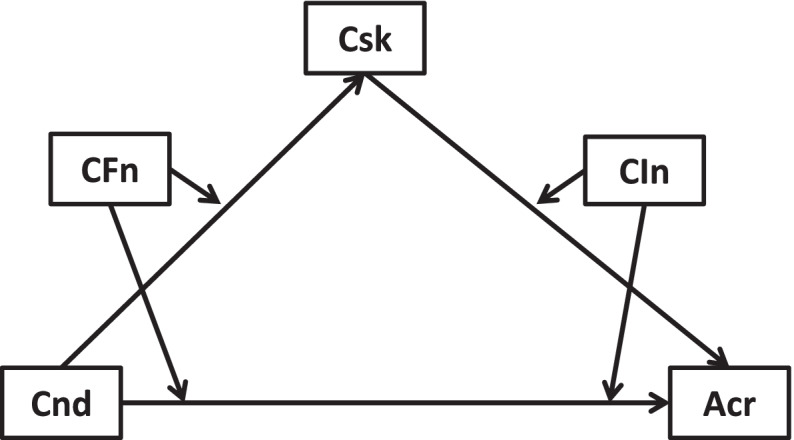
Cross-sectional link of new health conditions →accessible care mediated by COVID-19 risks and moderated by worse financial situation and lowered income

**Fig. 5:**
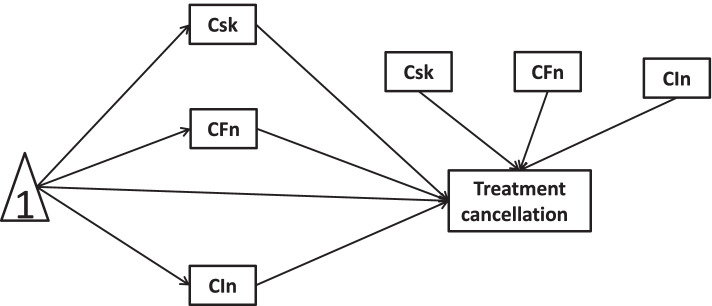
Longitudinal link of new health conditions →treatment cancellation mediated by COVID-19 risks, worse financial situation, and lowered income

**Fig. 6:**
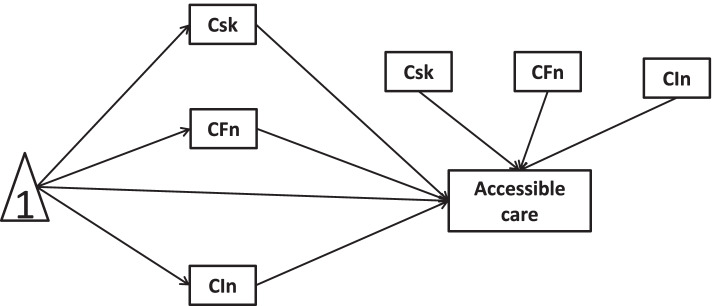
Longitudinal link of new health conditions →accessible care mediated by COVID-19 risks, worse financial situation, and lowered income

**Fig. 7:**
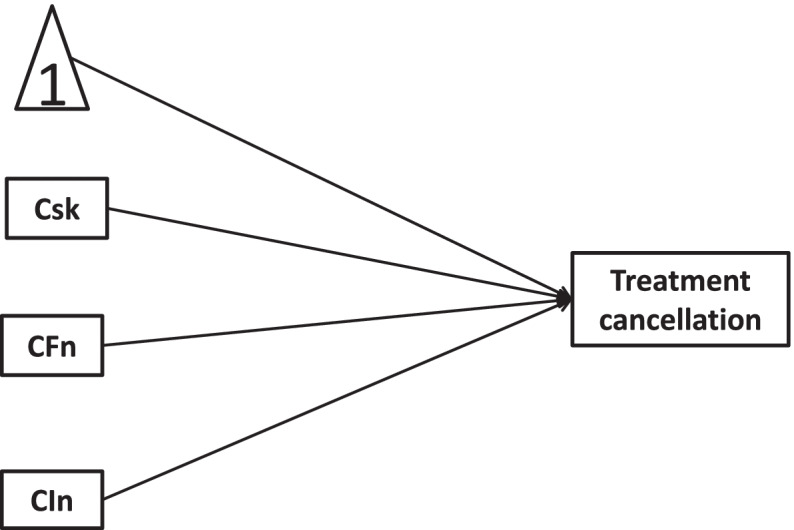
Longitudinal link of new health conditions →treatment cancellation moderated by COVID-19 risks, worse financial situation, and lowered income

**Fig. 8:**
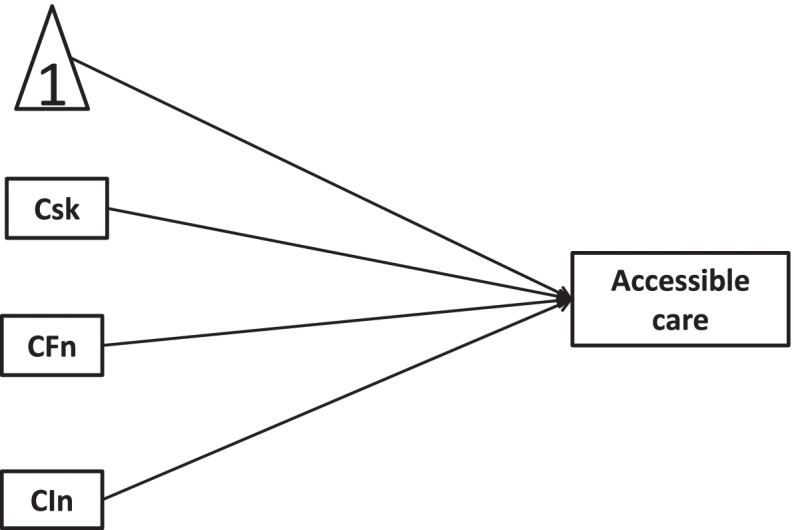
Longitudinal link of new health conditions →accessible care moderated by COVID-19 risks, worse financial situation, and lowered income

Here, this study employed publicly survey data to investigate the roles of COVID-19 risks, worse financial situation, and lowered income in the associations between new health conditions and healthcare service utilizations. Academically, mediation analysis is applied to quantify the causal sequence “predictor → mediators → outcome” for observational studies within path models. Moderation describes a situation in which the relationship “predictor → outcome” depends on the values of moderators. Since COVID-19 pandemic, multiple studies documented mediating/moderating roles of care/treatment in the relationship of interest. For example, a study treated healthcare service utilization as a mediating variable [65]. Another investigation treated new health condition as a mediating/moderating variable [66, 67]. Analogously, outcome variable in a similar research was consequent mental health status rather than healthcare service utilizations [68]. As compared to cross-sectional mediation/moderation, longitudinal mediation/moderation could reflect changes in mediating/moderating effects over time and lead to more accurate representations of the temporal precedence of change over time. Here, the conceptual frameworks of potential cross-sectional and longitudinal mediation/moderation are designed in Figs. 1, 2, 3, 4, 5, 6, 7 and 8.

In this study, we hypothesize that COVID-19 risks, worse financial situation, and lowered income mediate/moderate the relationship between new health conditions and healthcare service utilizations. To confirm the hypotheses of interest, the present study would quantify mediators and moderators with cross-sectional and longitudinal mediation/moderation models. The hypotheses of this study are as follows:

- H1a: The associations of new health conditions with treatment cancellation would be mediated by COVID-19 risks, worse financial situation, and lowered income in cross-sectional mediation models.
- H1b: The associations of new health conditions with accessible care would be mediated by COVID-19 risks, worse financial situation, and lowered income in cross-sectional mediation models.
- H2a: Worse financial situation and lowered income moderated the effect of COVID-19 risks on the associations of new health conditions with treatment cancellation in cross-sectional moderation models.
- H2b: Worse financial situation and lowered income moderated the effect of COVID-19 risks on the associations of new health conditions with accessible care in cross-sectional moderation models.
- H3a: There existed longitudinal mediation of COVID-19 risks, new health conditions, worse financial situations and lowered income on treatment cancellation.
- H3b: There existed longitudinal mediation of COVID-19 risks, new health conditions, worse financial situations and lowered income on accessible care.
- H4a: COVID-19 risks, new health conditions, and worse financial situations moderated associations of new health conditions with treatment cancellation in longitudinal moderation models.
- H4b: COVID-19 risks, new health conditions, and worse financial situations moderated associations of new health conditions with accessible care in longitudinal moderation models.

The empirical findings of this work would contribute to the related academic fields. Regarding theoretical implications, our study would extend prior research on the HBM application for the relationship between new health conditions and healthcare service utilizations with cross-sectional/longitudinal mediation/moderation. Considering practical implications, our study would provide new insights into health management among poverty-stricken older adults in the United Kingdom in the time of COVID-19.

## Methodology

### Data source

This study employed a survey-based data from The English Longitudinal Study of Ageing (ELSA) COVID-19 Substudy (https://www.elsa-project.ac.uk/covid-19). The ELSA COVID-19 Substudy allows a cross-sectional analysis of the dynamics of the lockdown, health changes, economic vulnerabilities, and health-seeking behaviors. This study has been reviewed and approved by the University College London Research Ethics Committee.

The ELSA sample is based on respondents aged 50 and over who participated in the Health Survey for England. The respondents from three years of 1998, 1999 and 2001 consisted of the original sample and provide a sufficiently large sample size (23,132 responding households). In the course of the study, the sample has refreshed at the younger age range to maintain the 50+ design.

The ELSA COVID-19 survey questionnaire covers the following topic areas: demographics, mental health, financial security, COVID-19-related health, employment and work, financial situation, volunteering and care, physical health and health behaviours, social connection isolation and technological inclusion, and income, pensions and retirement. The first and second wave of the substudy lasted 54 days (from the 3rd June 2020 to the 26th July 2020) and 47 days (from the 4th November 2020 to the 20th December 2020), respectively. The first wave of the ELSA COVID-19 Substudy aimed to understand the immediate impact of the COVID-19 crisis on health, access to health and social care, financial circumstances, mental wellbeing, and social activity in the older population in England. It also aimed to set a baseline for a second wave, which looked to assess what changes had taken place in mental and physical health, finances, and social experience of the older population by the end of 2020.

As for the ELSA COVID-19 Substudy, both cross-sectional and longitudinal weights were developed to maximize the available sample size and increase the statistical power of the analysis in conjunction with ELSA Wave 9. ELSA Wave 9 received ethical approval from the South Central – Berkshire Research Ethics Committee on 10th May 2018 (17/SC/0588). The cross-sectional weights in COVID-19 Substudy wave 1 included weight for core members only and for both core members and partners. The longitudinal weights in COVID-19 Substudy wave 2 were established for analyses of wave 1, wave 2, ELSA wave 9.Furthermore, given the relatively high response rate from partners, an additional weight was created to allow partners aged 52+ to be included in weighted analysis for COVID-19 Substudy waves 1 and 2.

Considering COVID-19 Substudy sampling approach, all participants for the COVID-19 substudy were selected from the existing ELSA sample. Study participants issued to wave 1 who were still eligible ahead of wave 2 and did not request to leave the COVID-19 Substudy or the ELSA study were issued to wave 2. Number of issued study participants was 9525 in wave 1 and 9150 in wave 2, respectively, while number of issued core members was 7689 in wave 1 and 7465 in wave 2, respectively. Number of completed survey interviews were 7040 in wave 1 and 6794 in wave 2, while number of productive core members were 5825 in wave 1 and 5338 in wave 2.With a sequential mixed-mode strategy (web + telephone) data collection, the ELSA COVID-19 Substudy achieved a final response rate of 75% (online: 83% and phone:17%).

ELSA is a so ripe and famous survey that it can guarantee complete confidentiality and avoid identification of individuals or organisations. All methods were carried out in accordance with relevant guidelines and regulations. Written informed consent was obtained from all participants before they agreed to participate in the study. Participants were informed that they could leave the study at any time without penalty, and all personal information was kept confidential.

### Main variables

Healthcare service included treatment cancellation and accessible care.

### Treatment cancellation

Treatment cancellation was reflected by the question: “Since the coronavirus outbreak, have you had a hospital operation or treatment cancelled?” with responses of no and yes in wave 1 and no, yes and “I did not have a hospital operation or treatment booked” in wave 2. The answer “I did not have a hospital operation or treatment booked” was treated as a missing value in wave 2. Thus, the response options were no (=0) and yes (=1).

### Accessible care

Accessible care were reflected by the question: “Since the coronavirus outbreak, have you been able to access the community health and social care services and support you need, for instance a dentist, podiatrist, nurse, counselling or personal care?” with responses of no, yes, “I did not attempt to contact them”, and “I did not need to contact them” in wave 1 and no, yes, and “I did not need to contact them” in wave 2. The answers “I did not attempt to contact them” and “I did not need to contact them” were treated as a missing value in waves 1 and 2. Thus, the response options were no (=0) and yes (=1).

### COVID-19 risks

Infected risk for COVID-19 consist of five questions: “Has anyone in your household tested positive for COVID-19?” , “Has anyone in your household had to stay in hospital for treatment due to COVID-19?” , “Has anyone close to you outside your household tested positive for COVID-19, for example a relative or a friend?”, “Has anyone close to you, such as a friend or family member, died with COVID-19?” , and “Has anyone in your household died with COVID-19?” Their response options were yes (=1) and no (=2). For convenience, missing values of the response options in the five questions are defined as zeros. Here, the response options were recoded as binary response (yes = 1, no = 0). Then, sum response options could be calculated and obtained COVID-19 risks.

### Disease exposure

#### New health conditions

New health conditions were reflected by the question: “Thinking about what has happened since we last saw you, has a doctor ever told you that you developed a new health condition?” The response options were High blood pressure or hypertension, Angina, a heart attack (including myocardial infarction or coronary thrombosis), Congestive heart failure, Diabetes or high blood sugar, A stroke (cerebral vascular disease), Chronic lung disease such as chronic bronchitis or emphysema, Asthma, Arthritis (including osteoarthritis, or rheumatism), Cancer or a malignant tumour (excluding minor skin cancers), Dementia, senility or another serious memory impairment, Alzheimer’s disease, and Malignant blood disorder, e.g. leukaemia. Number of new health conditions in wave 1 was distributed as 0 (80.11%), 1 (14.59%), 2 (3.58%), 3 (1.04%), 4 (0.39%), 5 (0.20%), 6 (0.07%), and 7 (0.03%), *N* = 6952. Number of new health conditions in wave 2 was distributed as 0 (83.34%), 1 (13.29%), 2 (2.31%), 3 (0.67%), 4 (0.25%), 5 (0.07%), and 6 (0.06%), *N* = 6710.

#### Economic vulnerability

Here, economic vulnerability included worse financial situations and lowered income.

#### Worse financial situation

Worse financial situation was reflected by the question: “How do you feel your current financial situation compares to before the coronavirus outbreak?” The response options were “I’m much worse off”, “I’m a little worse off”, “I’m about the same”, “I’m a little better off”, and “I’m much better off”. Thus, worse financial situation was recoded as no (=0, “I’m about the same”, “I’m a little better off”, and “I’m much better off”) and yes (=1, “I’m much worse off”, “I’m a little worse off”).

#### Lowered income

Lowered income was reflected by the question: “What is your current income level compared to before the coronavirus outbreak before the coronavirus outbreak that began in February?” The response options were higher, about the same, and lower. Here, lowered income was recoded as no (higher/about the same = 0) and yes (lower = 1).

#### Socio-demographical factors

Main socio-demographical factors included age (years), gender (male = 1, female = 0), and region (urban = 1, rural = 2) and long-standing condition. For convenience, age was subjectively divided by old group (50–64 years) and older group (65–84 years), oldest group (≥ 85 years). Long-standing condition was reflected by the question: “Do you have any long-standing illness, disability or infirmity?” with the response options of no (= 0) and yes (= 1).

### Statistical strategies

First, descriptive analyses were performed by gender in waves 1 and 2. Zero-inflated Poisson regression on COVID-19 risks and new health conditions were performed. Here, COVID-19 risks and new health conditions were count variables. Logistic regressions on healthcare service were examined under the condition that COVID-19 risks and new health conditions were categorized into no (=0) and yes (=1).

The study hypotheses from 1a to 2b were statistically similar to models 6 and 29 presented in PROCESS macro by reference [69] in supplementary Figs. 1 and 2, respectively. The conditional effect was tested based on a bias-corrected bootstrapping procedure with 5000 samples. A significant effect was signaled by a bootstrap confidence interval (95% CI) that does not include the “0” value. Some cases were deleted due to missing data. The moderating variables were mean centered prior to analysis.

Regarding cross-sectional mediation, the mediating effects of COVID-19 risks, worse financial situation, and lowered income on the relationships of “new health conditions → treatment cancellation” and “new health conditions → accessible care” in waves 1 and 2 were conducted by models 6 in Model templates for PROCESS v 2.16 for SPSS (http://www.guilford.com/p/hayes3). In Model 6, mediators 1, 2, and 3 were COVID-19 risks, worse financial situation, and lowered income, respectively.

Cross-sectional moderation models were conducted by model 29 in model templates for PROCESS v 2.16 for SPSS (http://www.guilford.com/p/hayes3). In Model 29, mediator was COVID-19 risks, W was worse financial situation, V was lowered income.

Longitudinal mediation and longitudinal moderation were conducted by models 1 and 2 in MEMORE (Mediation and Moderation for Repeated Measures; available at http://akmontoya.com), respectively. MEMORE procedure for SPSS version 2.1 was written by Amanda Montoya.

COVID-19 risks and new health conditions were continuous variables in cross-sectional mediation, cross-sectional moderation, longitudinal mediation, and longitudinal moderation. With respect to data cleaning, the participants for the ELSA COVID-19 Substudy with age < 50 were deleted. In this study, responses with “prefer not to say (-9)”, “don’t know (-8)”, “not asked (-6)”, “routing error (-3)”, and “item not applicable (-1)” were treated as missing values and excluded from the analysis. For this reason, the statistical analysis in this study was performed using original data without imputation.

## Results

### Descriptive analysis

Table 1 showed sample characteristics by gender in waves 1 and 2. There were significant gender differences in age groups, worse financial situation, and lowered income in waves 1 and 2. There were significant gender differences in treatment cancellation and accessible care in wave 1. Most of the sample was >65 years old (94.13%, *n* = 6952 in wave 1 vs. 94.31%, *n* = 6710 in wave 2), females (56.26%, *n* = 6952 in wave 1 vs. 56.11%, *n* = 6710 in wave 2), and located in urban place (72.53%, *n* = 6947 in wave 1 vs. 72.61%, *n* = 6695 in wave 2). The number involved in long-standing condition in wave 1 (52.99%, *n* = 6951) was higher than that in wave 2 (43.04%, *n* = 6705). 17.58% (*n* = 6952) faced COVID-19 risks in wave 1, while it was 24.10% (*n* = 6710) in wave 2. The number involved in worse financial situation in wave 1 (18.44%, *n* = 6946) was higher than that in in wave 2 (16.71%, *n* = 6701). With regard to income, 16.61% (*n* = 6930) in wave 1 and 14.09% (*n* = 6698) in wave 2 were lowered. 19.89% (*n* = 6952) suffered new health conditions in waves 1 and 2. In general, there was no statistical significance in gender among all the respondents between regions, COVID-19 risks, and new health conditions in waves 1 and 2. The proportion of treatment cancellation and accessible care was generally high. Some patients with specific health conditions could be at risk of being inappropriately treated due to COVID-19 pandemic.

**Table 1:** Sample characteristics by gender groups

	Wave 1			Wave 2		
	Female %	Male %	Chi square	Female %	Male %	Chi square
Age (N1 = 6952; N2 = 6710)			13.6175***			23.1389***
50–64	18.74	12.89		18.30	12.01	
65–84	34.11	28.39		34.55	29.45	
≥ 85	3.41	2.46		3.26	2.43	
Region (N1 = 6947; N2 = 6695)			1.0978			1.1421
Urban	41.08	31.45		41.00	31.61	
Rural	15.17	12.29		15.07	12.32	
Long-standing condition (N1 = 6951; N2 = 6705)			0.2008			1.5761
No	26.59	20.43		32.33	24.62	
Yes	29.68	23.31		23.77	19.27	
COVID-19 risks (N1 = 6952; N2 = 6710)			0.8526			0.4526
No	46.16	36.26		42.41	33.49	
Yes	10.10	7.48		13.70	10.40	
New health conditions (N1 = 6952; N2 = 6396)			0.8212			0.3729
No	44.85	35.26		45.34	35.57	
Yes	11.41	8.49		10.88	8.21	
Worse financial situation (N1 = 6946; N2 = 6701)			18.2710***			12.7434***
No	46.86	34.70		47.52	35.77	
Yes	9.39	9.06		8.57	8.15	
Lowered income (N1 = 6930; N2 = 6698)			6.6318**			6.5161**
No	47.42	35.97		48.69	37.22	
Yes	8.76	7.85		7.36	6.73	
Treatment cancellation (N1 = 6950; N2 = 3101)			6.4741**			0.0839
No	46.01	36.79		42.28	34.05	
Yes	10.24	6.95		13.25	10.42	
Accessible care (N1 = 1907; N2 = 3409)			5.3031**			2.6850
No	33.04	28.00		12.88	10.38	
Yes	23.18	15.78		45.00	31.74	

** and *** denote significance at 5, and 1% levels, respectively

### Factors associating with COVID-19 risks and new health conditions

In Table 2, older adults in age group 65–84 years were less likely to be involved in COVID-19 risks and new health conditions than adults in age group 50–64 years in waves 1 and 2. Older adults in age group ≥85 years were less likely to be involved in COVID-19 risks (wave 1) and new health conditions (waves 1 and 2) than adults in age group 50–64 years. Males had a lower probability of being involved in COVID-19 risks and new health conditions than females. Older adults with long-standing condition were more likely to be involved in new health conditions (in waves 1 and 2) and more likely to be involved in COVID-19 risks (in wave 2) than without. Older adults with lowered income were less likely to suffer new health conditions than those without in waves 1 and 2.

**Table 2:** Zero-inflated Poisson regression on new health conditions and COVID-19 risks, IRR (95% CI)

	Wave 1		Wave 2	
	New health conditions	COVID-19 risks	New health conditions	COVID-19 risks
Age	IRR (95% CI)	IRR (95% CI)	IRR (95% CI)	IRR (95% CI)
50–64	1[reference]	1[reference]	1[reference]	1[reference]
65–84	0.76*** (0.69, 0.84)	0.55*** (0.49, 0.61)	0.70*** (0.63, 0.77)	0.46*** (0.42, 0.51)
≥ 85	0.95(0.79, 1.14)	0.42*** (0.32, 0.57)	0.74** (0.58, 0.93)	0.38*** (0.30, 0.49)
Sex				
Female	1[reference]	1[reference]	1[reference]	1[reference]
Male	0.79*** (0.71, 0.87)	0.82*** (0.73, 0.91)	0.78*** (0.70, 0.88)	0.84*** (0.77, 0.92)
Long-standing condition				
No	1[reference]	1[reference]	1[reference]	1[reference]
Yes	1.48*** (1.35, 1.63)	0.96(0.86, 1.06)	1.37*** (1.23, 1.52)	0.90** (0.82, 0.99)
Worse financial situation				
No	1[reference]	1[reference]	1[reference]	1[reference]
Yes	0.88(0.76, 1.03)	0.94(0.80, 1.11)	0.96(0.80, 1.15)	0.95(0.82, 1.10)
Lowered income				
No	1[reference]	1[reference]	1[reference]	1[reference]
Yes	0.79*** (0.67, 0.93)	1.01(0.86, 1.20)	0.80** (0.65, 0.97)	0.93(0.80, 1.09)
inflate				
region	0.20** (0.04, 0.36)	0.10(−0.08, 0.29)	0.18** (0.01, 0.36)	0.47 *** (0.25, 0.69)
Constant	3.72*** (0.20, 0.64)	0.30** (0.04, 0.57)	0.58*** (0.34, 0.83)	−0.97 *** (−1.30, −0.63)
Number	6921	6921	6674	6674

*IRR* incidence relative risk, *95% CI* 95% confidence interval

** and *** denote significance at 5, and 1% levels, respectively

### Factors associating with healthcare service

In Table 3, older adults in age group 65–84 years had lower probability of treatment cancellation in waves 1 and 2 and accessible care in wave 1 and higher probability of accessible care in wave 2 as compared with older adults in age group 50–64 years. Compared with older adults in age group 50–64 years, older adults in age group ≥85 years had lower probability of treatment cancellation in waves 1 and 2 and higher probability of accessible care in wave 2. Compared with females, males had lower probability of treatment cancellation in waves 1 and 2 and accessible care in wave 1 and higher probability of accessible care in wave 2. Compared with older adults in urban place, older adults in rural place had lower probability of treatment cancellation in waves 1 and 2 and higher probability of accessible care in wave 2. Older adults with long-standing condition were more likely to cancel treatment and access care than without (in wave 2). Compared with older adults without coviod-19 risk, older adults with coviod-19 risk had lower probability of treatment cancellation in waves 1 and 2 and higher probability of accessible care in wave 2. Older adults with condition were more likely to cancel treatment and access care than without, while older adults with financial were less likely to cancel treatment and access care than without (in wave 1). Older adults with lowered income were less likely to cancel treatment (in wave 1) and more likely to access care than without (in wave 2).

**Table 3:** Logistic regressions on healthcare service utilizations, AOR (95% CI)

	Wave 1				Wave 2			
	Treatment cancellation		Accessible care		Treatment cancellation		Accessible care	
Age	AOR	95% CI	AOR	95% CI	AOR	95% CI	AOR	95% CI
50–64	1[reference]	1[reference]	1[reference]	1[reference]	1[reference]	1[reference]	1[reference]	1[reference]
65–84	0.38***	0.34, 0.42	0.79***	0.66, 0.94	0.40***	0.34, 0.46	2.79***	2.42, 3.21
≥85	0.41***	0.32, 0.53	1.02	0.73, 1.42	0.53***	0.37, 0.74	2.58***	1.77, 3.77
Sex								
Female	1[reference]	1[reference]	1[reference]	1[reference]	1[reference]	1[reference]	1[reference]	1[reference]
Male	0.46***	0.41, 0.52	0.72***	0.60, 0.87	0.63***	0.54, 0.75	1.15*	0.98, 1.35
Region								
Urban	1[reference]	1[reference]	1[reference]	1[reference]	1[reference]	1[reference]	1[reference]	1[reference]
Rural	0.57***	0.50, 0.65	1.04	0.85, 1.28	0.65***	0.53, 0.78	1.57***	1.30, 1.88
Long-standing condition								
No	1[reference]	1[reference]	1[reference]	1[reference]	1[reference]	1[reference]	1[reference]	1[reference]
Yes	0.95	0.85, 1.06	0.91	0.76, 1.09	1.31***	1.11, 1.54	1.19**	1.01, 1.39
COVID-19 risks								
No	1[reference]	1[reference]	1[reference]	1[reference]	1[reference]	1[reference]	1[reference]	1[reference]
Yes	0.69 ***	0.59, 0.81	0.84	0.67, 1.07	0.72***	0.60, 0.86	1.38***	1.15, 1.65
New health conditions								
No	1[reference]	1[reference]	1[reference]	1[reference]	1[reference]	1[reference]	1[reference]	1[reference]
Yes	1.46***	1.26, 1.68	1.45***	1.18, 1.78	1.14	0.94, 1.39	0.98	0.80, 1.20
Worse financial situation								
No	1[reference]	1[reference]	1[reference]	1[reference]	1[reference]	1[reference]	1[reference]	1[reference]
Yes	0.82**	0.68, 0.98	0.62***	0.47, 0.82	0.85	0.66, 1.10	0.87	0.68, 1.11
Lowered income								
No	1[reference]	1[reference]	1[reference]	1[reference]	1[reference]	1[reference]	1[reference]	1[reference]
Yes	0.55***	0.45, 0.67	0.84	0.63, 1.13	0.81	0.61, 1.07	1.27*	0.98, 1.66
Number	6921		1895		2903		3237	

*AOR* Adjusted odds ratio, *95% CI* 95% confidence interval

*, **, *** denote significance at 10, 5, and 1% levels, respectively

### Cross-sectional mediation

In Table 4, there were no significant indirect effect(s) of COVID-19 risks, worse financial situation, and lowered income on the relationship of new health conditions → treatment cancellation in wave 1. But, there were significant indirect effect(s) of COVID-19 risks, worse financial situation, and lowered income on the relationship of new health conditions →treatment cancellation in wave 2. Thus, H1a was partially accepted. Simultaneously, there were no significant indirect effect(s) of COVID-19 risks, worse financial situation, and lowered income on the relationship of new health conditions →accessible care in waves 1 and 2. Mediating coefficients of COVID-19 risks (Csk), worse financial situation (CFn), and lowered income (CIn) on new health conditions (Cnd) → treatment cancellation (Tcn) and Cnd → accessible care (Acr) could be found in Supplementary Tables 1, 2, 3 and 4. Thus, H1b was rejected.

**Table 4:** Cross-sectional mediations

Total effect model	Wave 1						Wave 2					
	Cnd → Tcn			Cnd → Acr			Cnd → Tcn			Cnd → Acr		
	Coeff.	LLCI	ULCI	Coeff.	LLCI	ULCI	Coeff.	LLCI	ULCI	Coeff.	LLCI	ULCI
constant	−1.7471	−1.8178	−1.6765	−.5205	−.6236	−.4173	−1.2113	−1.3029	−1.1197	1.2250	1.1383	1.3118
Cnd	.4965	.4178	.5751	.1665	.0594	.2736	.1293	.0088	.2497	−.1132	−.2302	.0038
-2LL	6199.0070			2525.2782			3376.7636			3684.6879		
Model LL	149.3664			9.3217			4.2735			3.4770		
McFadden	.0235			.0037			.0013			.0009		
CoxSnell	.0213			.0049			.0014			.0010		
Nagelkrk	.0356			.0067			.0021			.0015		
	Effect	LLCI	ULCI	Effect	LLCI	ULCI	Effect	LLCI	ULCI	Effect	LLCI	ULCI
Total effect of X on Y	.4965	.4178	.5751	.1665	.0594	.2736	.1293	.0088	.2497	−.1132	−.2302	.0038
Direct effect of X on Y	.4972	.4183	.5760	.1647	.0573	.2721	.1196	−.0015	.2406	−.1022	−.2203	.0159
Indirect effect(s) of X on Y												
Total	.0008	−.0035	.0064				.0099	.0008	.0254			
Cnd - > Csk - > Tcn	.0001	−.0030	.0040				.0073	.0010	.0199			
Cnd - > Csk - > CFn- > Tcn	.0000	−.0001	.0001				.0001	−.0001	.0006			
Cnd - > Csk - > CIn - > Tcn	.0000	−.0001	.0000				.0001	.0000	.0008			
Cnd - > Csk - > CFn- > CIn - > Tcn	.0000	.0000	.0000				.0001	.0000	.0004			
Cnd - > CFn- > Tcn	−.0003	−.0042	.0016				.0011	−.0031	.0096			
Cnd - > CFn- > CIn- > Tcn	.0001	−.0004	.0012				.0012	−.0005	.0057			
Cnd - > CIn - > Tcn	.0009	−.0012	.0053				.0001	−.0033	.0046			
Indirect effect(s) of X on Y												
Total				.0026	−.0074	.0143				−.0113	−.0299	.0026
Cnd - > Csk - > Acr				−.0004	−.0065	.0050				−.0055	−.0159	−.0004
Cnd - > Csk- > CFn- > Acr				.0002	.0000	.0010				−.0005	−.0018	.0000
Cnd - > Csk- > CIn- > Acr				.0001	.0000	.0005				.0000	−.0002	.0002
Cnd - > Csk - > CFn - > CIn - > Acr				.0000	.0000	.0003				.0000	−.0002	.0001
Cnd - > CFn- > Acr				.0010	−.0051	.0097				−.0053	−.0189	.0058
Cnd- > CFn- > CIn- > Acr				.0002	−.0008	.0033				.0000	−.0022	.0016
Cnd- > CIn - > Acr				.0015	−.0014	.0101				.0000	−.0024	.0028
N	6926			1896			3090			3399		

*Cnd* new health conditions, *Tcn* treatment cancellation, *Acr* accessible care, *Csk* COVID-19 risks, *CFn* worse financial situation, *CIn* = lowered income, *LLCI* low limit confidence interval, *ULCI* upper limit confidence interval

### Cross-sectional moderation

In Table 5, the coefficients of Cnd × CFn, Csk × CIn, Cnd × CFn, and Cnd × CIn were not significant in wave 1. Thus, there were no moderating effects of worse financial situation and lowered income on the mediating COVID-19 risks on the associations of new health conditions with treatment cancellation. Likewise, there were no moderating effects of worse financial situation and lowered income on the mediating COVID-19 risks on the associations of new health conditions with accessible care. Furthermore, the coefficients of Cnd × CFn were significant in wave 2. But, the coefficients of Csk × CIn, Cnd × CFn, Cnd × CIn were not significant. Thus, there were moderating effects of worse financial situation on the Cnd → Csk. Accordingly, H2a and H2b were rejected.

**Table 5:** Cross-sectional moderations

	Wave 1						Wave 2					
	Cnd → Tcn			Cnd → Acr			Cnd → Tcn			Cnd → Acr		
	Csk			Csk			Csk			Csk		
	Coeff.	LLCI	ULCI	Coeff.	LLCI	ULCI	Coeff.	LLCI	ULCI	Coeff.	LLCI	ULCI
Constant	.2091	.1947	.2235	.2339	.2051	.2626	−.0008	−.0207	.0192	−.0005	−.0198	.0187
Cnd	.0015	−.0182	.0212	−.0183	−.0489	.0122	.0341	.0033	.0649	.0316	.0016	.0617
CFn	.0530	.0198	.0862	.0408	−.0240	.1057	−.0280	−.0548	−.0012	−.0291	−.0542	−.0040
Cnd × CFn	−.0031	−.0487	.0425	−.0470	−.1142	.0203	−.0467	−.0835	−.0099	−.0587	−.0945	−.0229
R-sq	.0016			.0034			.0054			.0062		
	Tcn			Acr			Tcn			Acr		
Constant	−1.8084	−1.8951	−1.7216	−.4118	−.5372	−.2865	−1.1799	−1.2634	−1.0964	1.2068	1.1263	1.2874
Csk	.1860	.0543	.3178	−.0338	−.2357	.1681	.1817	.0401	.3232	−.1631	−.2975	−.0286
Cnd	.4943	.4042	.5844	.1413	.0180	.2646	.1098	−.0141	.2337	−.1077	−.2270	.0115
CIn1	−.0472	−.2824	.1880	−.2159	−.5718	.1400	.1216	−.0846	.3279	−.0147	−.2144	.1850
Csk × CIn	−.0500	−.3386	.2386	.2372	−.2143	.6887	.0305	−.2424	.3035	.1320	−.1318	.3958
CFn1	.1451	−.0610	.3511	−.4126	−.7318	−.0934	−.0270	−.1511	.0972	.2646	.1447	.3844
Cnd × CFn	−.0906	−.3130	.1319	−.0109	−.3137	.2920	−.0704	−.2303	.0894	−.0212	−.1770	.1345
Cnd × CIn	.1284	−.1101	.3669	.1826	−.1512	.5164	.0865	−.2152	.3881	.0054	−.2910	.3019
-2LL	6187.6329			2508.8552			3364.9685			3652.6888		
Model LL	160.7405			25.7447			16.0686			35.4761		
McFadden	.0253			.0102			.0048			.0096		
CoxSnell	.0229			.0135			.0052			.0104		
Nagelkrk	.0382			.0183			.0078			.0157		
N	6926			1896			3090			3399		

*Cnd* new health conditions, *Tcn* treatment cancellation, *Acr* accessible care, *Csk* COVID-19 risks, *CFn* worse financial situation, *CIn* lowered income, *LLCI* low limit confidence interval, *ULCI* upper limit confidence interval

### Longitudinal mediation

As presented in Table 6, effect coefficients of ‘X’ → (Tcn1-Tcn2)(β = −.0451, *p* < .0001, LLCI = -.0618, ULCI = -.0284), ‘X’ → (Csk1-Csk2) (β = .0592, *p* < .0001, LLCI = .0361, ULCI = .0824), and ‘X’ → (CIn1-CIn2) (β = −.0351, *p* < .0001, LLCI = -.0523, ULCI = -.0179) were significant. Thus, ‘X’ → (Tcn1-Tcn2), ‘X’ → (Csk1-Csk2), and ‘X’ → (CIn1-CIn2) existed. Additionally, effect coefficients of ‘X’ → (Acr2- Acr1) (β = .3687, *p* < .0001, LLCI = .3350, ULCI = .4025), ‘X’ → (Csk2-Csk1) (β = .0676, *p* = .0005, LLCI = .0294, ULCI = .1058), and ‘X’ → (CIn1-CIn2) (β = −.0369, *p* = .0102, LLCI = -.0650, ULCI = -.0087) were significant. Thus, ‘X’ → (Acr2- Acr1), ‘X’ → (Csk2-Csk1), and ‘X’ → (CIn1-CIn2) existed. But, indirect effect of coefficients of X on Y through M in the analyses of ‘X’ → treatment cancellation and accessible care were not significant. Consequently, H3a and H3b were rejected.

**Table 6:** Longitudinal mediations

	Treatment cancellation						Accessible care				
	Effect	SE	p	LLCI	ULCI		Effect	SE	p	LLCI	ULCI
Tcn2-Tcn1						Acr2- Acr1					
‘X’	−.0451	.0085	.0000	−.0618	−.0284	‘X’	.3687	.0172	.0000	.3350	.4025
Csk1-Csk2						Csk1-Csk2					
‘X’	.0592	.0118	.0000	.0361	.0824	‘X’	.0676	.0195	.0005	.0294	.1058
CFn1-CFn2						CFn1-CFn2					
‘X’	.0145	.0143	.3132	−.0137	.0426	‘X’	.0105	.0235	.6536	−.0355	.0566
CIn1-CIn2						CIn1-CIn2					
‘X’	−.0351	.0088	.0001	−.0523	−.0179	‘X’	−.0369	.0143	.0102	−.0650	−.0087
	coeff	SE	p	LLCI	ULCI		coeff	SE	p	LLCI	ULCI
‘X’	−.0460	.0086	.0000	−.0629	−.0292	‘X’	.3701	.0173	.0000	.3362	.4041
M1diff	.0211	.0134	.1171	−.0053	.0474	M1diff	−.0025	.0263	.9237	−.0540	.0490
M2diff	.0009	.0113	.9363	−.0213	.0231	M2diff	.0631	.0225	.0051	.0190	.1072
M3diff	.0096	.0185	.6031	−.0266	.0459	M3diff	.0513	.0369	.1648	−.0211	.1237
M1avg	.0250	.0191	.1915	−.0125	.0626	M1avg	.0281	.0379	.4583	−.0462	.1025
M2avg	.0184	.0152	.2245	−.0113	.0482	M2avg	.0314	.0307	.3069	−.0289	.0917
M3avg	−.0058	.0255	.8207	−.0557	.0442	M3avg	−.0020	.0519	.9690	−.1038	.0998
Total effect of X on Y						Total effect of X on Y					
	Effect	SE	p	LLCI	ULCI		Effect	SE	p	LLCI	ULCI
	−.0451	.0085	.0000	−.0618	−.0284		.3687	.0172	.0000	.3350	.4025
Direct effect of X on Y						Direct effect of X on Y					
	Effect	SE	p	LLCI	ULCI		Effect	SE	p	LLCI	ULCI
	−.0460	.0086	.0000	−.0629	−.0292		.3701	.0173	.0000	.3362	.4041
Indirect Effect of X on Y through M						Indirect Effect of X on Y through M					
		Effect	BootSE	BootLLCI	BootULCI			Effect	BootSE	BootLLCI	BootULCI
	Ind11	.0012	.0008	−.0002	.0031		Ind12	−.0002	.0018	−.0037	.0036
	Ind21	.0000	.0002	−.0004	.0006		Ind22	.0007	.0016	−.0021	.0044
	Ind31	−.0003	.0007	−.0017	.0010		Ind32	−.0019	.0016	−.0063	.0003
	Total	.0009	.0011	−.0011	.0032		Total	−.0014	.0028	−.0071	.0041
R-sq	.0024						.0088				
N	2903						1139				

*Tcn1* treatment cancellation in wave 1, *Tcn2* treatment cancellation in wave 2, *Acr1* accessible care in wave 1, *Acr2* accessible care in wave 2, *Csk1* COVID-19 risks in wave 1, *Csk2* COVID-19 risks in wave 2, *CFn1* worse financial situation in wave 1, *CFn2* worse financial situation in wave 2, *CIn1* lowered income in wave 1, *CIn2* lowered income in wave 2

Ydiff1 = Tcn2- Tcn1, Ydiff2 = Acr2-Acr1, M1diff = Csk2-Csk1, M2diff = CFn2-CFn1, M3diff = CInc2-CInc1, M1avg = (Cvrisk2 + Crisk1)/2, M2avg = (CFn2 + CFn1)/2, M3avg = (CInc2 + CInc1)/2, Ind11 = ‘X’- > M1diff- > Ydiff1, Ind21 = ‘X’- > M2diff- > Ydiff1, Ind31 = ‘X’- > M3diff- > Ydiff1, Ind12 = ‘X’- > M1diff- > Ydiff2, Ind22 = ‘X’- > M2diff- > Ydiff2, Ind32 = ‘X’- > M3diff- > Ydiff2. LLCI = low limit confidence interval. ULCI = upper limit confidence interval

### Longitudinal moderation

As presented in Table 7, Csk2 could significantly predict Tcn2-Tcn1 (β = .0337, *p* = .0357, LLCI = .0023, ULCI = .0652 in Model 1 and β = .0328, *p* = .0412, LLCI = .0013, ULCI = .0643 in Model 2) and Tcn2 (β = 0341, *p* = .0219, LLCI = .0049, ULCI = .0633 in Model 1 and β = .0342, *p* = .0217, LLCI = .0050, ULCI = .0634 in Model 2) in wave 2. Additionally, CFn2 could significantly predict Tcn1 (β = −.0292, *p* = .0218, LLCI = -.0541, ULCI = -.0043) in wave 1. CFn2 could significantly predict Acr2-Acr1 (β = .0708, *p* = .0056, LLCI = .0208, ULCI = .1208 in Model 3) and Acr2 (β = .0584, *p* = .0031, LLCI = .0198, ULCI = .0971 in Model 2) in wave 2. Thus, H4a and H4b were rejected.

**Table 7:** Longitudinal moderations

	Model 1				Model 2				Model 3				Model 4			
	β	P	LLCI	ULCI	β	P	LLCI	ULCI	β	P	LLCI	ULCI	β	P	LLCI	ULCI
	Tcn2-Tcn1								Acr2- Acr1							
const	−.1170	.0040	−.1965	−.0374	−.0067	.8869	−.0985	.0851	.2625	.0012	.1035	.4215	.4234	.0000	.2365	.6104
Csk2	.0337	.0357	.0023	.0652	.0328	.0412	.0013	.0643	.0095	.7577	−.0508	.0698	.0117	.7047	−.0491	.0726
Csk1	−.0088	.6045	−.0419	.0244	−.0090	.5937	−.0422	.0241	.0189	.5717	−.0468	.0846	.0137	.6851	−.0527	.0802
CFn2	.0109	.4006	−.0145	.0363					.0708	.0056	.0208	.1208				
CFn1	.0104	.4077	−.0143	.0351					−.0378	.1302	−.0867	.0112				
CIn2					−.0021	.9217	−.0439	.0397					.0085	.8441	−.0760	.0929
CIn1					−.0196	.3479	−.0605	.0213					−.0373	.3632	−.1177	.0431
R^2^	.0024				.0018				.0071				.0011			
	Tcn2								Acr2							
const	.2472	.0000	.1734	.3209	.2029	.0000	.1178	.2879	.5482	.0000	.4253	.6710	.8162	.0000	.6714	.9609
Csk2	.0341	.0219	.0049	.0633	.0342	.0217	.0050	.0634	−.0043	.8580	−.0509	.0424	−.0069	.7734	−.0540	.0402
Csk1	.0102	.5134	−.0205	.0409	.0104	.5069	−.0203	.0411	.0012	.9626	−.0496	.0520	−.0029	.9105	−.0544	.0485
CFn2	−.0183	.1281	−.0419	.0053					.0584	.0031	.0198	.0971				
CFn1	.0109	.3503	−.0120	.0338					.0000	1.0000	−.0378	.0378				
CIn2					.0320	.1050	−.0067	.0708					−.0229	.4922	−.0882	.0425
CIn1					−.0212	.2719	−.0591	.0166					−.0214	.5009	−.0836	.0409
R^2^	.0034				.0036				.0099				.0016			
	Tcn1								Acr1							
const	.3641	.0000	.2861	.4422	.2095	.0000	.1195	.2995	.2856	.0000	.1543	.4170	.3927	.0000	.2389	.5466
Csk2	.0004	.9803	−.0305	.0313	.0014	.9306	−.0295	.0323	−.0137	.5886	−.0636	.0361	−.0187	.4644	−.0687	.0314
Csk1	.0190	.2520	−.0135	.0515	.0194	.2417	−.0131	.0519	−.0177	.5216	−.0720	.0365	−.0167	.5494	−.0713	.0380
CFn2	−.0292	.0218	−.0541	−.0043					−.0123	.5588	−.0536	.0290				
CFn1	.0005	.9692	−.0238	.0247					.0378	.0670	−.0027	.0782				
CIn2					.0341	.1028	−.0069	.0752					−.0313	.3761	−.1008	.0381
CIn1					−.0016	.9357	−.0417	.0384					.0159	.6368	−.0502	.0821
R^2^	.0029				.0017				.0041			.0019				
N	2913				2907				1149				1139			

constant = constant, Csk1 = COVID-19 risks in wave 1, Csk2 = COVID-19 risks in wave 2, CFn1 = worse financial situation in wave 1, CFn2 = worse financial situation in wave 2, and CIn1 = lowered income in wave 1, CIn2 = lowered income in wave 2

*LLCI* low limit confidence interval, *ULCI* upper limit confidence interval

## Discussion

The proportion of treatment cancellation and accessible care in wave 2 was higher than those in wave 1. Empirically, zero-inflated Poisson regression and logistic regression indicated COVID-19 risks, worse financial situation, and lowered income were associated with treatment cancellation and accessible care. Obviously, older adults with worse financial situation and lowered income had a considerably less likelihood of treatment cancellation and accessible care in wave 1 rather than 2. The current research found the effects of new health conditions on treatment cancellation and accessible care indirectly through worse financial situation, lowered income and COVID-19 risks existed in longitudinal mediation models rather than cross-sectional mediation models, cross-sectional moderation models, and longitudinal moderation models. Thus, COVID-19 risks, worse financial situation, and lowered income were most identified as barriers to healthcare in this study.

The results in this study were in line with the findings in a study among adult Israeli Jews. The study reported adults aged ≥60+ years and reporting higher levels of COVID-19 fear were more likely to report forgone care than younger and less concerned adults during the COVID-19 lockdown period [70]. Likewise, COVID-19-associated delays in seeking care were reported in Antananarivo, Madagascar [71]. Similarly, a cross-sectional study indicated that the COVID-19 pandemic was associated with reductions in hospitalizations for ambulatory care-sensitive conditions [72].

The findings from longitudinal mediation could be partially explained by some studies. Serious adverse consequences accompanied by COVID-19 restrictions are reported in the field of health management. Inaccessible health care among adults with diabetes during the COVID-19 pandemic was documented in the United States [73]. COVID-19 lockdowns lead to declines in health care utilization. It is necessary to equip community health care services amid COVID-19 [74]. Likewise, insignificant cross-sectional mediation models, cross-sectional moderation models, and longitudinal moderation could also be explained by findings from international comparison study. The study indicated negative financial effects on health care providers in the England were not severe than that in the United States during COVID-19 pandemic [75].

The findings in this study were in line with the mismatch between health care demand and supply reported in early studies. For example, there were high disproportionate risks for COVID-19 hospitalization pressures varied by spatial and socio-demographic factors in England and Wales [76]. A qualitative study found that fear of COVID-19 was a major factor to hinder access to maternal healthcare services during the onset of the COVID-19 pandemic in Kenya [77]. A Chinese study indicated social factors and type of healthcare utilization were associated with the time delay for diagnosis [78]. Furthermore, an observational cohort study indicated worse health and function were partial determinants of more hospital admissions among people aged ≥50 years with lower wealth [79]. Also, a repeated cross-sectional study indicated older adults experiencing financial hardship had markedly greater risks of mental disorders after accounting for COVID-19 social assistance receipt [80].

This research is helpful in gaining a better understanding of the relationship between health service needs and health care provision. The role of economic conditions was explanatory in line with findings in prior studies. In details, unmet need in health care use may relate to individuals’ ability to pay [81]. Additionally, poorer access to care might be the determinants of higher levels of unmet social needs among low-income adults [82]. Especially, low-income individuals with serious health conditions have poorer access to outpatient care than their higher income counterparts [83].

Some conjecture may be correct. COVID-19 risks, worse financial situation and lowered income must exacerbate adults with long-standing condition and new health conditions face accessing health care. Some people with long-standing condition and new health conditions are at great risk of contracting COVID-19 because they require health and social care and are unable to access. Additionally, some people with worse financial situation and lowered income have COVID-19 risks if they become infected. Despite this, UK governments have recognized and responded to diverse health care needs of people with long-standing condition, new health conditions, worse financial situation and lowered income during COVID-19 pandemic.

### Policy implications

As for policy practice, the findings of the current study indicated older adults at risk for experiencing worse financial situation and lowered income could be targeted to participate in interventions to reduce their levels of COVID-19 risks. The fundamental role of COVID-19 risks as a mediator makes it evident that reduction in COVID-19 risks can promote health-seeking behaviors. The findings stress the importance of developing interventions aimed at mitigating COVID-19 risks, worse financial situation and lowered income while consuming healthcare services. In the short term, healthcare services need to reduce COVID-19 risks under conditions of uncertain demand. In the long term, policymakers should consider alternative ways of delivering healthcare services to the adults with worse financial situation and lowered income during crisis. Therefore, there is a need to redesign the strategies of health care service during COVID-19 pandemic. Additionally, it is necessary to strengthen primary health care services, particularly by diminishing obstacles for groups with worse financial situation and lowered income.

Persons with preexisting health conditions or disabilities may be worse due to the COVID-19 pandemic controlling strategies. A study reported that disruption to care caused by COVID-19 or insufficient quality of the new telemedicine care could lead to psychological disorders which was mediated by self-efficacy [84]. Early screening, recognition, diagnoses, and treatment of health conditions can prevent complications, improve quality of life, and help reduce health care costs. Untreated medical conditions affect mortality, functional disability, quality of life, and health care costs, especially for the uninsured individuals. Part of patients with specific health conditions could be at risk of being inappropriately treated during the COVID-19 pandemic. Family members can play in supporting patients’ self-care. MHealth can reduce barriers to health care access and facilitate integrated health care models for vulnerable populations [85]. With respect to indirect impact of the COVID-19 pandemic on the health-care system, a retrospective cohort study indicated undiagnosed conditions or delayed diagnosis of health conditions increased substantially [86].

### Limitations

Some limitations of the present study should be noted. First, a short-term longitudinal study with two waves could not draw accurate causal conclusions. Second, some key variables like lockdown, lifestyle and treatment cost were not surveyed in the dataset used in this study. Similarly, some key variables like medical counseling in wave 1 did not appear in wave 2. Particularly, limited to the topic of interest, some vital independent variables were not inserted into regression models. For example, ageing can make adults frail and depend on transport to health access of the elderly. But, there were no indirect association between age and health care use to report in this study. Finally, low response rates in possibly limited the generalization of the empirical findings. Thus, the findings in this study cannot be generalised to the wider communities in these countries.

### Strengths

First, mediation and moderation analyses were performed after regression screen out potential mediators and moderators. Second, mediation and moderation analyses of COVID-19 risks, new health conditions, and worse financial situations on the relationships of new health conditions → treatment cancellation and new health conditions → accessible care were conducted using the PROCESS tool and MEMORE procedure.

### Future research directions

First, association of functional status with treatment cancellation and accessible care need to be explored in the future. Second, association of mental status with treatment cancellation and accessible care may be new direction in the future. This is can be reflected by several studies which reported the association between COVID-19-related financial situation and mental health [87, 88]. If replicating in future studies, it is recommended that research with a longitudinal design be undertaken irrespective of crisis settings. Moreover, more research is needed to explore the associations of interest with other mediators and moderators like transportation barriers.

## Conclusions

In summary, the findings in longitudinal mediation models reveal the potential influence of worse financial situation, lowered income and COVID-19 risks and their relevance for future research. There were not significant mediating and moderating worse financial situation, lowered income and COVID-19 risks in the relationships: new health conditions → treatment cancellation and new health conditions → accessible care. The COVID-19 risk negatively mediated treatment cancellation and accessible care, while worse financial situation and lowered income positively mediated them.

## Supplementary Information

**Additional file 1.**

**Additional file 2: Supplementary table 1**. Mediating coefficients of Csk1, CFn1, and CIn1 on the link of Cnd1→Tcn1 in Model 6 (*N*= 6926). **Supplementary table 2**. Mediating coefficients of Csk1, CFn1, and CIn1 on the link of Cnd1→Acr11 in Model 6 (*N*=1896). **Supplementary table 3**. Mediating coefficients of Csk2, CFn2, and CIn2 on the link of Cnd2→Tcn2 in Model 6 (*N*= 3090). **Supplementary table 4**. Mediating coefficients of Csk2, CFn2, and CIn2 on the link of Cnd2→Acr2 in Model 6 (*N*=3399).

## Data Availability

Access to the survey data is open and publicly available in the following link. https://www.elsa-project.ac.uk/covid-19

## Abbreviations

COVID-19: Coronavirus disease-19
IRR: Incidence relative risk
95% CI: 95% confidence interval
Csk: COVID-19 risks
CFn: Worse financial situation
CIn: Lowered income
Cnd: New health conditions
Tcn: Treatment cancellation
Acr: Accessible care
LLCI: Low limit confidence interval
ULCI: Upper limit confidence interval
