# Supplementary material for: Associations between new health conditions and healthcare service utilizations among older adults in the United Kingdom: effects of COVID-19 risks, worse financial situation, and lowered income
Source: BMC Geriatr. 2022 Apr 22;22:356. doi: 10.1186/s12877-022-02995-8 (PMC9030688; doi:10.1186/s12877-022-02995-8)
Supplement: Supplementary file 1 — Additional file 1. [file 12877_2022_2995_MOESM1_ESM.pptx]

## Slide 1
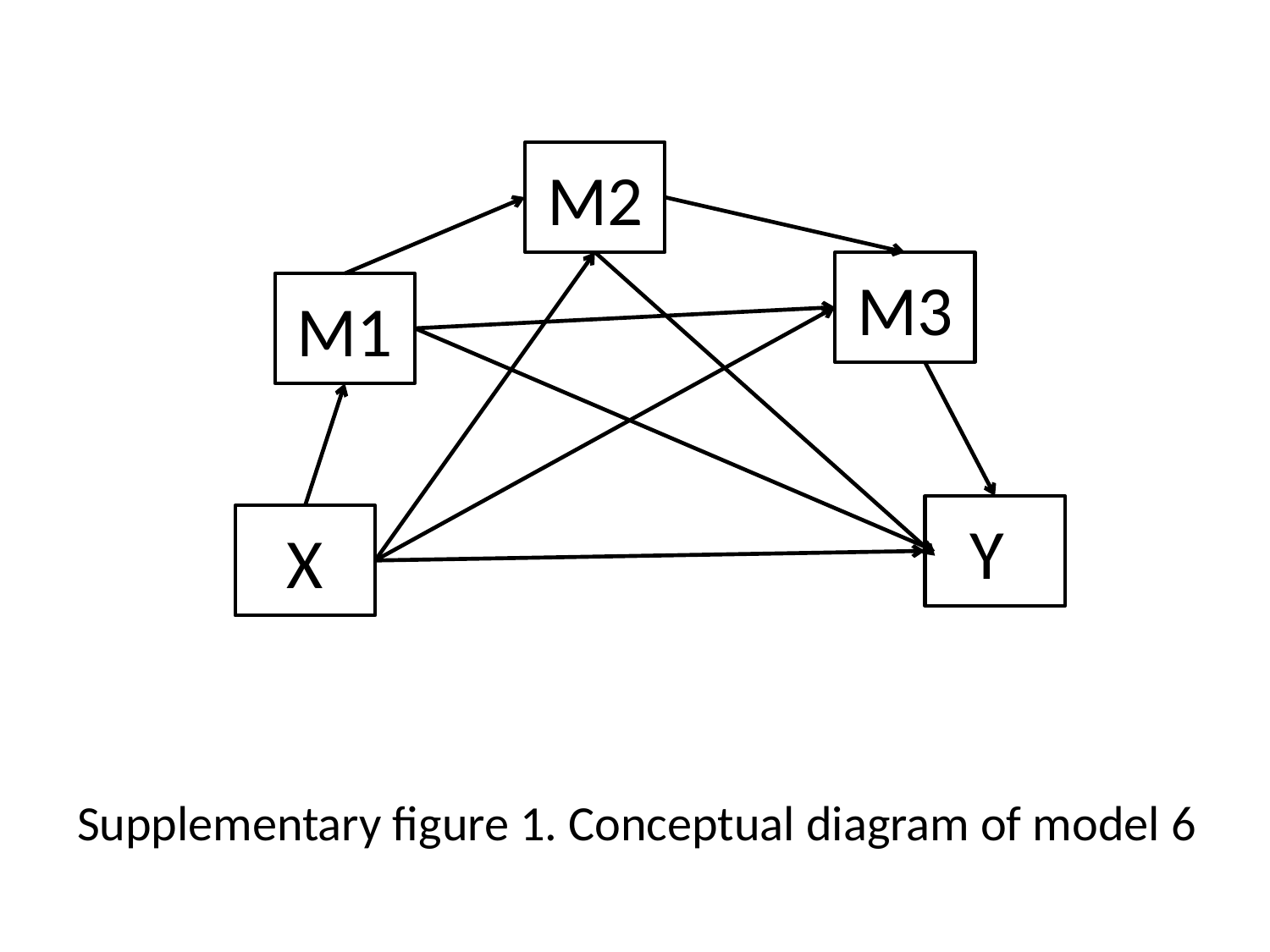

M2
M3
M1
Y
X
Supplementary figure 1. Conceptual diagram of model 6

## Slide 2
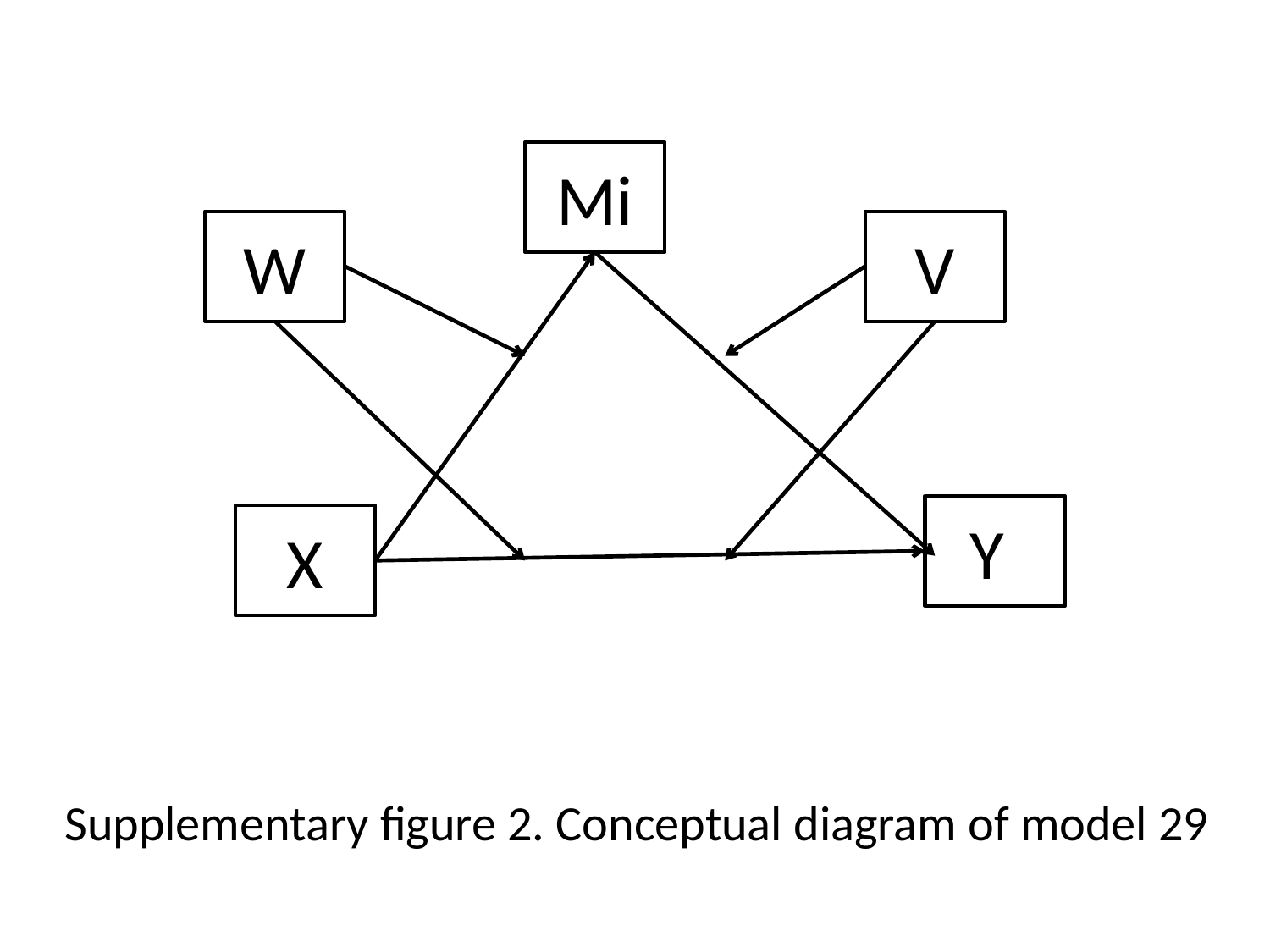

Mi
W
V
Y
X
Supplementary figure 2. Conceptual diagram of model 29
