# Supplementary material for: Associations between new health conditions and healthcare service utilizations among older adults in the United Kingdom: effects of COVID-19 risks, worse financial situation, and lowered income
Source: BMC Geriatr. 2022 Apr 22;22:356. doi: 10.1186/s12877-022-02995-8 (PMC9030688; doi:10.1186/s12877-022-02995-8)
Supplement: Supplementary file 2 — Additional file 2: Supplementary table 1. Mediating coefficients of Csk1, CFn1, and CIn1 on the link of Cnd1→Tcn1 in Model 6 (N= 6926). Supplementary table 2. Mediating coefficients of Csk1, CFn1, and CIn1 on the link of Cnd1→Acr11 in Model 6 (N=1896). Supplementary table 3. Mediating coefficients of Csk2, CFn2, and CIn2 on the link of Cnd2→Tcn2 in Model 6 (N= 3090). Supplementary table 4. Mediating coefficients of Csk2, CFn2, and CIn2 on the link of Cnd2→Acr2 in Model 6 (N=3399). [file 12877_2022_2995_MOESM2_ESM.docx]

Supplementary table 1. Mediating coefficients of Csk1, CFn1, and CIn1 on the link of Cnd1→Tcn1 in Model 6 (N= 6926).

| Csk1 |  | coeff | se | t | p | LLCI | ULCI |
| --- | --- | --- | --- | --- | --- | --- | --- |
|  | constant | .2190 | .0066 | 33.0822 | .0000 | .2060 | .2320 |
|  | Cnd1 | .0006 | .0091 | .0621 | .9505 | -.0172 | .0183 |
|  | R-sq | .0000 |  |  |  |  |  |
| CFn1 |  | coeff | se | t | p | LLCI | ULCI |
|  | constant | 3.0195 | .0107 | 283.3737 | .0000 | 2.9986 | 3.0404 |
|  | Csk1 | -.0467 | .0180 | -2.5966 | .0094 | -.0819 | -.0114 |
|  | Cnd1 | .0041 | .0136 | .3042 | .7610 | -.0225 | .0307 |
|  | R-sq | .0010 |  |  |  |  |  |
| CIn1 |  | coeff | se | t | p | LLCI | ULCI |
|  | constant | 2.8905 | .0204 | 141.8466 | .0000 | 2.8506 | 2.9305 |
|  | Csk1 | .0293 | .0097 | 3.0274 | .0025 | .0103 | .0483 |
|  | CFn1 | -.2593 | .0065 | -40.0509 | .0000 | -.2720 | -.2467 |
|  | Cnd1 | -.0160 | .0073 | -2.1917 | .0284 | -.0303 | -.0017 |
|  | R-sq | .1904 |  |  |  |  |  |
| Tcn1 |  | coeff | se | Z | p | LLCI | ULCI |
|  | constant | -1.4253 | .2634 | -5.4111 | .0000 | -1.9416 | -.9091 |
|  | Csk1 | .1759 | .0598 | 2.9407 | .0033 | .0587 | .2932 |
|  | CFn1 | -.0800 | .0473 | -1.6906 | .0909 | -.1728 | .0128 |
|  | CIn1 | -.0584 | .0785 | -.7439 | .4569 | -.2123 | .0955 |
|  | Cnd1 | .4972 | .0402 | 12.3598 | .0000 | .4183 | .5760 |
|  | -2LL | 6187.6889 |  |  |  |  |  |
|  | Model LL | 160.6845 |  |  |  |  |  |
|  | McFadden | .0253 |  |  |  |  |  |
|  | CoxSnell | .0229 |  |  |  |  |  |
|  | Nagelkrk | .0382 |  |  |  |  |  |

Note: Csk1= COVID-19 risks in wave 1, CFn1= worse financial situation in wave 1, CIn1= lowered income in wave 1, Tcn1= treatment cancellation in wave 1, and Cnd1 = new health conditions in wave 1. LLCI= low limit confidence interval. ULCI= upper limit confidence interval.

Supplementary table 2. Mediating coefficients of Csk1, CFn1, and CIn1 on the link of Cnd1→Acr11 in Model 6 (N=1896).

| Csk1 |  | coeff | se | t | p | LLCI | ULCI |
| --- | --- | --- | --- | --- | --- | --- | --- |
|  | constant | .2420 | .0131 | 18.4140 | .0000 | .2162 | .2678 |
|  | Cnd1 | -.0280 | .0139 | -2.0154 | .0440 | -.0552 | -.0008 |
|  | R-sq | .0021 |  |  |  |  |  |
| CFn1 |  |  |  |  |  |  |  |
|  | constant | 2.9991 | .0213 | 140.9534 | .0000 | 2.9574 | 3.0409 |
|  | Csk1 | -.0538 | .0343 | -1.5705 | .1165 | -.1210 | .0134 |
|  | Cnd1 | .0066 | .0207 | .3202 | .7488 | -.0340 | .0473 |
|  | R-sq | .0014 |  |  |  |  |  |
| CIn1 |  |  |  |  |  |  |  |
|  | constant | 2.9104 | .0390 | 74.6132 | .0000 | 2.8339 | 2.9869 |
|  | Csk1 | .0207 | .0185 | 1.1152 | .2649 | -.0157 | .0570 |
|  | CFn1 | -.2652 | .0124 | -21.3374 | .0000 | -.2895 | -.2408 |
|  | Cnd1 | -.0157 | .0112 | -1.4004 | .1616 | -.0377 | .0063 |
|  | R-sq | .1961 |  |  |  |  |  |
| Acr1 |  |  |  |  |  |  |  |
|  | constant | -.7708 | .3835 | -2.0098 | .0445 | -1.5225 | -.0191 |
|  | Csk1 | .0152 | .0919 | .1655 | .8686 | -.1650 | .1954 |
|  | CFn1 | .1512 | .0688 | 2.1993 | .0279 | .0165 | .2860 |
|  | CIn1 | -.0977 | .1143 | -.8550 | .3926 | -.3218 | .1263 |
|  | Cnd1 | .1647 | .0548 | 3.0051 | .0027 | .0573 | .2721 |
|  | -2LL | 2516.3901 |  |  |  |  |  |
|  | Model LL | 18.2098 |  |  |  |  |  |
|  | McFadden | .0072 |  |  |  |  |  |
|  | CoxSnell | .0096 |  |  |  |  |  |
|  | Nagelkrk | .0130 |  |  |  |  |  |

Note: Csk1= COVID-19 risks in wave 1, CFn1= worse financial situation in wave 1, CIn1= lowered income in wave 1, Cnd1 = new health conditions in wave 1, and Acr1 = accessible care in wave 1. LLCI= low limit confidence interval. ULCI= upper limit confidence interval.

Supplementary table 3. Mediating coefficients of Csk2, CFn2, and CIn2 on the link of Cnd2→Tcn2 in Model 6 (N= 3090).

| Csk2 |  | coeff | se | t | p | LLCI | ULCI |
| --- | --- | --- | --- | --- | --- | --- | --- |
|  | constant | .2952 | .0112 | 26.4019 | .0000 | .2733 | .3171 |
|  | Cnd2 | .0383 | .0157 | 2.4451 | .0145 | .0076 | .0691 |
|  | R-sq | .0019 |  |  |  |  |  |
| CFn2 |  | coeff | se | t | p | LLCI | ULCI |
|  | constant | 3.0264 | .0163 | 185.9803 | .0000 | 2.9945 | 3.0583 |
|  | Csk2 | -.0505 | .0237 | -2.1352 | .0328 | -.0969 | -.0041 |
|  | Cnd2 | -.0372 | .0206 | -1.8005 | .0719 | -.0776 | .0033 |
|  | R-sq | .0026 |  |  |  |  |  |
| CIn2 |  | coeff | se | t | p | LLCI | ULCI |
|  | constant | 2.8587 | .0310 | 92.1254 | .0000 | 2.7978 | 2.9195 |
|  | Csk2 | .0196 | .0129 | 1.5185 | .1290 | -.0057 | .0450 |
|  | CFn2 | -.2634 | .0098 | -26.8085 | .0000 | -.2826 | -.2441 |
|  | Cnd2 | .0006 | .0113 | .0531 | .9576 | -.0215 | .0227 |
|  | R-sq | .1905 |  |  |  |  |  |
| Tcn2 |  | coeff | se | Z | p | LLCI | ULCI |
|  | constant | -1.4358 | .3484 | -4.1216 | .0000 | -2.1186 | -.7530 |
|  | Csk2 | .1894 | .0715 | 2.6477 | .0081 | .0492 | .3296 |
|  | CFn2 | -.0308 | .0630 | -.4893 | .6247 | -.1544 | .0927 |
|  | CIn2 | .1241 | .1039 | 1.1937 | .2326 | -.0796 | .3278 |
|  | Cnd2 | .1196 | .0617 | 1.9364 | .0528 | -.0015 | .2406 |
|  | -2LL | 3366.7892 |  |  |  |  |  |
|  | Model LL | 14.2479 |  |  |  |  |  |
|  | McFadden | .0042 |  |  |  |  |  |
|  | CoxSnell | .0046 |  |  |  |  |  |
|  | Nagelkrk | .0069 |  |  |  |  |  |

Note: Csk2= COVID-19 risks in wave 2, CFn2= worse financial situation in wave 2, CIn2= lowered income in wave 2, Tcn2= treatment cancellation in wave 2, and Cnd2 = new health conditions in wave 2. LLCI= low limit confidence interval. ULCI= upper limit confidence interval.

Supplementary table 4. Mediating coefficients of Csk2, CFn2, and CIn2 on the link of Cnd2→Acr2 in Model 6 (N=3399).

| Csk2 |  | coeff | se | t | p | LLCI | ULCI |
| --- | --- | --- | --- | --- | --- | --- | --- |
|  | constant | .3008 | .0107 | 28.1771 | .0000 | .2799 | .3217 |
|  | Cnd2 | .0355 | .0153 | 2.3161 | .0206 | .0054 | .0655 |
|  | R-sq | .0016 |  |  |  |  |  |
| CFn2 |  |  |  |  |  |  |  |
|  | constant | 3.0246 | .0159 | 190.6708 | .0000 | 2.9935 | 3.0557 |
|  | Csk2 | -.0540 | .0230 | -2.3532 | .0187 | -.0990 | -.0090 |
|  | Cnd2 | -.0203 | .0205 | -.9900 | .3222 | -.0605 | .0199 |
|  | R-sq | .0020 |  |  |  |  |  |
| CIn2 |  |  |  |  |  |  |  |
|  | constant | 2.9151 | .0284 | 102.5585 | .0000 | 2.8594 | 2.9709 |
|  | Csk2 | .0156 | .0120 | 1.2932 | .1960 | -.0080 | .0391 |
|  | CFn2 | -.2779 | .0090 | -30.9206 | .0000 | -.2955 | -.2603 |
|  | Cnd2 | -.0055 | .0107 | -.5151 | .6065 | -.0266 | .0155 |
|  | R-sq | .2209 |  |  |  |  |  |
| Acr2 |  |  |  |  |  |  |  |
|  | constant | .4994 | .3415 | 1.4621 | .1437 | -.1701 | 1.1688 |
|  | Csk2 | -.1550 | .0683 | -2.2689 | .0233 | -.2889 | -.0211 |
|  | CFn2 | .2630 | .0610 | 4.3135 | .0000 | .1435 | .3825 |
|  | CIn2 | -.0027 | .1010 | -.0272 | .9783 | -.2007 | .1952 |
|  | Cnd2 | -.1022 | .0602 | -1.6959 | .0899 | -.2203 | .0159 |
|  | -2LL | 3653.7900 |  |  |  |  |  |
|  | Model LL | 34.3749 |  |  |  |  |  |
|  | McFadden | .0093 |  |  |  |  |  |
|  | CoxSnell | .0101 |  |  |  |  |  |
|  | Nagelkrk | .0152 |  |  |  |  |  |

Note: Csk2= COVID-19 risks in wave 2, CFn2= worse financial situation in wave 2, CIn2= lowered income in wave 2, Cnd2 = new health conditions in wave 2, and Acr2 = accessible care in wave 2. LLCI= low limit confidence interval. ULCI= upper limit confidence interval.
